# Supplementary material for: A Novel Effector FlSp1 Inhibits the Colonization of Endophytic Fusarium lateritium and Increases the Resistance to Ralstonia solanacearum in Tobacco
Source: J Fungi (Basel). 2023 Apr 27;9(5):519. doi: 10.3390/jof9050519 (PMC10219502; doi:10.3390/jof9050519)
Supplement: Supplementary file 1 [file jof-09-00519-s001.zip › jof-2297268-supplementary.pdf]

Supplementary data

Article Title：A novel effector *FlSp1* inhibits the colonization of endophytic *Fusarium lateritium* and increases the resistance to *Ralstonia solanacearum* in tobacco

Supplementary table 1. Prediction and proportion of effectors.

Supplementary table 2. According to BLAST comparison of *F. lateritium,* 69 effectors are characterized proteins.

Supplementary Figure 1. Knockout of *FlSp1* by homologous recombination strategy.

Supplementary Figure 2. *FlSp1* causes plant immune response but not leaf death.

Supplementary Figure 3. *FlSp1* does not affect tobacco growth.

Supplementary Table S1. Prediction and proportion of effectors

Protein type	Number	(%)Percent
Signal peptide	1502	10.17
Transmembrane protein	3114	21.1
Secreted protein	1139	7.7
Effector	213	1.4

Supplementary Table S2. According to BLAST comparison of *F. lateritium,* 69 effectors are characterized proteins

Number	Gene number	(aa)Length	Effector probability	Annotation
1	EVM0006333.1	177	0.844	uncharacterized
2	EVM0009636.1	176	0.643	uncharacterized
3	EVM0005471.1	246	0.648	uncharacterized
4	EVM0013370.1	318	0.581	uncharacterized
5	EVM0008733.1	108	0.638	uncharacterized
6	EVM0014116.1	193	0.67	uncharacterized
7	EVM0003410.1	137	0.695	uncharacterized
8	EVM0000423.1	102	0.786	uncharacterized

9	EVM0008355.1	270	0.624	uncharacterized
10	EVM0006157.1	85	0.571	uncharacterized
11	EVM0013819.1	170	0.603	uncharacterized
12	EVM0012882.2	196	0.75	characterized
13	EVM0010567.1	304	0.648	uncharacterized
14	EVM0002215.2	260	0.563	characterized
15	EVM0007040.1	65	0.91	uncharacterized
16	EVM0001139.1	83	0.715	uncharacterized
17	EVM0009597.1	189	0.66	uncharacterized
18	EVM0013376.1	131	0.912	uncharacterized
19	EVM0012156.1	219	0.86	uncharacterized
20	EVM0010414.1	186	0.824	characterized
21	EVM0001349.1	220	0.777	characterized
22	EVM0007796.1	81	0.896	uncharacterized
23	EVM0012723.1	278	0.581	uncharacterized
24	EVM0012779.1	125	0.879	uncharacterized
25	EVM0010980.1	184	0.756	characterized
26	EVM0012042.1	306	0.565	characterized
27	EVM0009179.1	324	0.567	characterized
28	EVM0005640.1	240	0.679	characterized
29	EVM0011395.1	148	0.909	uncharacterized
30	EVM0002480.1	96	0.952	uncharacterized
31	EVM0002922.1	147	0.794	characterized
32	EVM0004658.1	220	0.779	uncharacterized
33	EVM0006367.1	226	0.699	uncharacterized
34	EVM0006057.1	96	0.898	uncharacterized
35	EVM0014039.1	129	0.826	uncharacterized
36	EVM0001932.1	274	0.557	characterized
37	EVM0004347.1	151	0.783	uncharacterized
38	EVM0007344.1	235	0.738	uncharacterized
39	EVM0004957.1	98	0.948	uncharacterized
40	EVM0007201.1	457	0.559	uncharacterized
41	EVM0001500.1	226	0.647	uncharacterized
42	EVM0011667.1	162	0.63	uncharacterized
43	EVM0001735.1	147	0.58	uncharacterized
44	EVM0005322.1	350	0.606	characterized
45	EVM0012487.1	237	0.828	uncharacterized
46	EVM0014208.1	195	0.953	characterized
47	EVM0010877.1	129	0.846	uncharacterized
48	EVM0012954.1	164	0.847	uncharacterized
49	EVM0011510.1	149	0.899	uncharacterized
50	EVM0001120.1	141	0.825	uncharacterized
51	EVM0014610.1	319	0.682	uncharacterized
52	EVM0001443.1	97	0.952	uncharacterized

53	EVM0000057.1	322	0.555	uncharacterized
54	EVM0001142.1	88	0.943	uncharacterized
55	EVM0007002.1	97	0.965	characterized
56	EVM0014586.1	126	0.739	uncharacterized
57	EVM0004505.1	243	0.676	characterized
58	EVM0003108.1	224	0.675	characterized
59	EVM0014328.1	221	0.787	characterized
60	EVM0006138.1	120	0.886	uncharacterized
61	EVM0014498.1	199	0.867	uncharacterized
62	EVM0014140.1	295	0.648	characterized
63	EVM0000294.1	378	0.597	characterized
64	EVM0002123.1	69	0.876	characterized
65	EVM0014671.1	175	0.583	uncharacterized
66	EVM0001162.1	155	0.869	uncharacterized
67	EVM0013657.1	322	0.613	characterized
68	EVM0005907.1	105	0.928	uncharacterized
69	EVM0001402.1	169	0.577	uncharacterized
70	EVM0005281.1	241	0.867	characterized
71	EVM0009167.1	166	0.611	uncharacterized
72	EVM0005763.1	219	0.631	characterized
73	EVM0003868.1	165	0.858	uncharacterized
74	EVM0008887.1	138	0.877	characterized
75	EVM0013489.1	245	0.623	uncharacterized
76	EVM0012583.1	101	0.965	characterized
77	EVM0003173.1	375	0.622	characterized
78	EVM0009206.1	239	0.76	characterized
79	EVM0003594.1	148	0.81	uncharacterized
80	EVM0007246.1	92	0.555	uncharacterized
81	EVM0012131.1	52	0.857	uncharacterized
82	EVM0006172.1	237	0.867	characterized
83	EVM0007284.1	208	0.802	uncharacterized
84	EVM0001143.1	154	0.823	characterized
85	EVM0011315.1	257	0.683	characterized
86	EVM0012488.1	233	0.887	characterized
87	EVM0010962.2	183	0.872	uncharacterized
88	EVM0002011.1	227	0.637	uncharacterized
89	EVM0009437.1	396	0.698	uncharacterized
90	EVM0013980.1	328	0.572	characterized
91	EVM0001537.1	210	0.69	uncharacterized
92	EVM0005253.1	121	0.959	uncharacterized
93	EVM0005607.1	70	0.657	uncharacterized
94	EVM0006239.1	240	0.841	characterized
95	EVM0009658.1	359	0.589	characterized
96	EVM0011051.1	108	0.979	uncharacterized

97	EVM0010473.1	203	0.775	uncharacterized
98	EVM0012330.1	232	0.925	characterized
99	EVM0006836.1	247	0.79	uncharacterized
100	EVM0013359.1	362	0.551	characterized
101	EVM0005836.1	263	0.616	characterized
102	EVM0004362.1	239	0.938	uncharacterized
103	EVM0010244.1	284	0.576	uncharacterized
104	EVM0008735.1	203	0.807	uncharacterized
105	EVM0007467.1	217	0.67	uncharacterized
106	EVM0006213.1	294	0.574	uncharacterized
107	EVM0013638.1	207	0.777	uncharacterized
108	EVM0004519.1	265	0.664	uncharacterized
109	EVM0014131.1	247	0.613	uncharacterized
110	EVM0012687.1	233	0.586	uncharacterized
111	EVM0002974.1	202	0.615	uncharacterized
112	EVM0000091.1	103	0.69	uncharacterized
113	EVM0007433.1	139	0.632	characterized
114	EVM0004198.1	170	0.81	uncharacterized
115	EVM0004696.1	123	0.86	characterized
116	EVM0000945.1	210	0.637	uncharacterized
117	EVM0007829.1	310	0.602	uncharacterized
118	EVM0009670.1	190	0.673	uncharacterized
119	EVM0003288.1	271	0.715	uncharacterized
120	EVM0014406.1	298	0.603	uncharacterized
121	EVM0002867.1	135	0.85	uncharacterized
122	EVM0004583.1	129	0.879	uncharacterized
123	EVM0004199.1	133	0.876	uncharacterized
124	EVM0005146.1	138	0.813	uncharacterized
125	EVM0010327.1	129	0.894	uncharacterized
126	EVM0012892.1	339	0.804	characterized
127	EVM0008124.1	218	0.864	characterized
128	EVM0001445.1	140	0.909	uncharacterized
129	EVM0007948.1	186	0.681	characterized
130	EVM0014535.1	169	0.667	uncharacterized
131	EVM0012415.1	239	0.638	uncharacterized
132	EVM0005891.1	224	0.91	characterized
133	EVM0005605.1	159	0.692	uncharacterized
134	EVM0014710.1	266	0.661	characterized
135	EVM0012557.1	270	0.572	uncharacterized
136	EVM0013223.1	124	0.704	uncharacterized
137	EVM0003335.1	166	0.637	uncharacterized
138	EVM0000889.1	183	0.869	uncharacterized
139	EVM0010402.1	100	0.815	uncharacterized
140	EVM0012384.1	217	0.726	uncharacterized

141	EVM0000095.1	257	0.748	uncharacterized
142	EVM0006154.1	103	0.715	uncharacterized
143	EVM0011042.1	125	0.816	uncharacterized
144	EVM0009850.1	213	0.873	characterized
145	EVM0006803.1	113	0.589	characterized
146	EVM0009679.1	126	0.814	characterized
147	EVM0011201.1	263	0.619	characterized
148	EVM0003773.1	171	0.815	uncharacterized
149	EVM0002915.1	144	0.667	uncharacterized
150	EVM0003054.1	149	0.585	uncharacterized
151	EVM0000104.1	89	0.952	uncharacterized
152	EVM0012618.1	85	0.795	uncharacterized
153	EVM0007631.1	114	0.756	uncharacterized
154	EVM0013159.1	154	0.66	characterized
155	EVM0012550.1	319	0.656	characterized
156	EVM0000795.1	192	0.708	uncharacterized
157	EVM0008422.1	252	0.662	uncharacterized
158	EVM0004881.1	338	0.713	characterized
159	EVM0012941.1	114	0.86	uncharacterized
160	EVM0009692.1	287	0.604	uncharacterized
161	EVM0008930.1	123	0.906	uncharacterized
162	EVM0013634.1	132	0.854	uncharacterized
163	EVM0004563.1	147	0.94	uncharacterized
164	EVM0010345.1	231	0.594	characterized
165	EVM0014036.1	230	0.584	characterized
166	EVM0005533.1	181	0.788	uncharacterized
167	EVM0000223.1	180	0.741	uncharacterized
168	EVM0004854.1	184	0.669	uncharacterized
169	EVM0007488.1	355	0.712	characterized
170	EVM0004195.1	242	0.742	characterized
171	EVM0012907.1	148	0.606	uncharacterized
172	EVM0008347.1	254	0.822	characterized
173	EVM0003893.1	181	0.61	uncharacterized
174	EVM0002443.1	330	0.616	uncharacterized
175	EVM0011077.1	292	0.594	uncharacterized
176	EVM0006417.1	138	0.615	uncharacterized
177	EVM0009264.1	142	0.802	characterized
178	EVM0013140.1	247	0.706	characterized
179	EVM0005593.1	468	0.568	characterized
180	EVM0005092.1	235	0.572	uncharacterized
181	EVM0009607.1	147	0.672	characterized
182	EVM0010696.1	191	0.788	uncharacterized
183	EVM0008599.2	239	0.856	characterized
184	EVM0002391.1	230	0.883	uncharacterized

185	EVM0003581.1	89	0.551	uncharacterized
186	EVM0004871.1	253	0.828	characterized
187	EVM0013936.1	123	0.811	uncharacterized
188	EVM0000278.1	126	0.839	characterized
189	EVM0004856.1	127	0.768	uncharacterized
190	EVM0005781.1	152	0.972	uncharacterized
191	EVM0007080.1	122	0.81	uncharacterized
192	EVM0008550.1	298	0.551	characterized
193	EVM0005978.1	251	0.581	characterized
194	EVM0003690.1	265	0.571	uncharacterized
195	EVM0008652.1	113	0.838	uncharacterized
196	EVM0003861.1	94	0.966	uncharacterized
197	EVM0006459.1	115	0.868	uncharacterized
198	EVM0013983.1	266	0.609	uncharacterized
199	EVM0013145.1	236	0.857	uncharacterized
200	EVM0000497.1	241	0.902	characterized
201	EVM0011643.1	231	0.717	characterized
202	EVM0004101.1	106	0.906	uncharacterized
203	EVM0013655.1	204	0.855	characterized
204	EVM0013306.1	63	0.77	uncharacterized
205	EVM0009868.1	137	0.944	uncharacterized
206	EVM0001748.1	170	0.76	uncharacterized
207	EVM0003744.1	71	0.769	uncharacterized
208	EVM0001829.1	145	0.921	uncharacterized
209	EVM0011186.1	144	0.931	uncharacterized
210	EVM0010618.1	98	0.769	characterized
211	EVM0000822.1	259	0.6	characterized
212	EVM0014202.1	127	0.971	uncharacterized
213	EVM0005809.1	124	0.949	uncharacterized

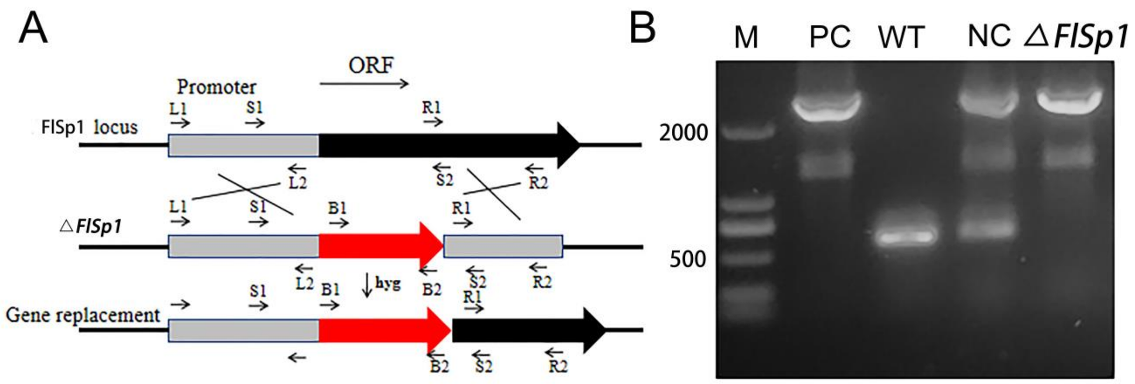

Supplementary Figure S1. Knockout of *FlSp1* by homologous recombination strategy. (A) Schematic representation of the replacement of *FlSp1* sequence by homologous recombination with GDPA+hyg fragment (1778 bp). (B) Validation of *FlSp1* knockout transformants. By designing primers at 260 bp position upstream and downstream of the FlS1 gene sequence

and performing PCR amplification, the transformants with unsuccessful homologous recombination could amplify a bright band of about 570 bp, and the transformants with normal knockdown of *FlSp1* could amplify a bright band of about 2348 bp.

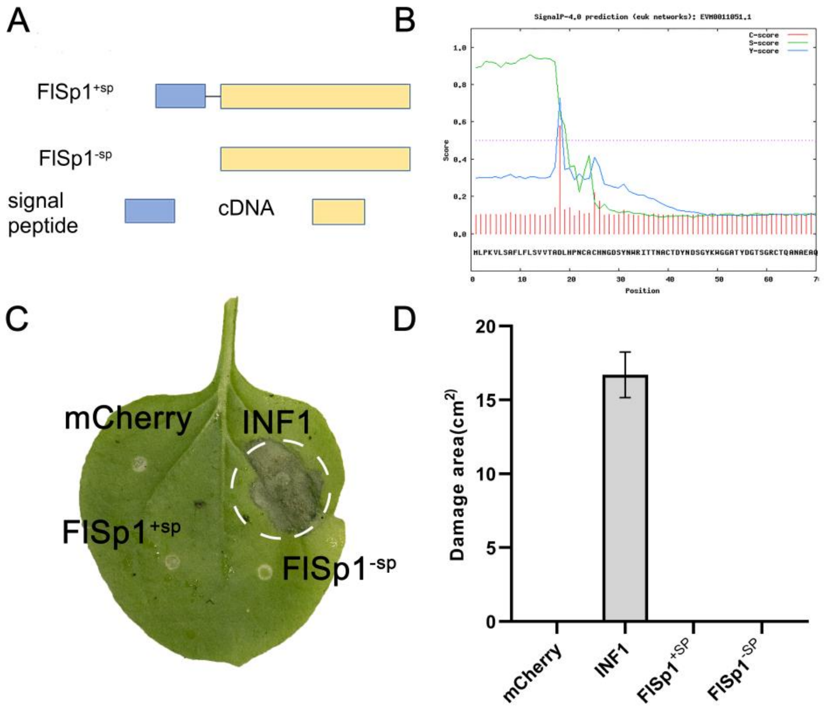

Supplementary Figure S2. *FlSp1* causes plant immune response but not leaf death. (A-B) Schematic diagram of *FlSp1* amino acid sequence. The blue area indicates the signal peptide position, which is at 1-17 aa (C) Neither *FlSp1*$^{+sp}$ nor *FlSp1*$^{-sp}$ caused death in plant leaves. mCherry is the negative control and INF1 is the positive control (D) Statistics of leaf death area after transient expression.

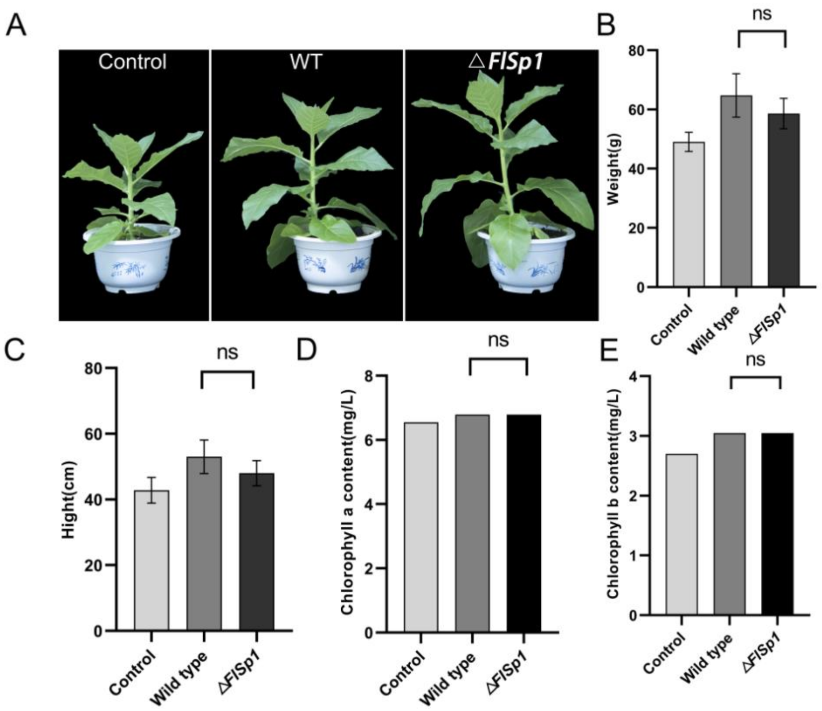

Supplementary Figure S3. *FlSp1* does not affect tobacco growth. (A) *FlSP1* does not affect the growth of plants. (B-E) After 14 d of incubation, plant fresh weight, plant height, and chlorophyll a and chlorophyll b contained in leaves were counted for each treatment. Eight replicates were set up for each experiment, and three experiments were conducted.
